# Effects of a Nutrition Education Programme in Stage IV Cardiac Rehabilitation Patients: A 3-Arm Randomised Controlled Trial

**DOI:** 10.3390/life14010063

**Published:** 2023-12-29

**Authors:** Jonathan Sinclair, Stephanie Dillon, Nicola M. Lowe, April Melia

**Affiliations:** 1 Centre for Global Development, School of Health, Social Work & Sport, University of Central Lancashire, Preston PR1 2HE, Lancashire, UK; 2 Research Centre for Applied Sport, Physical Activity and Performance, School of Health, Social Work & Sport, University of Central Lancashire, Preston PR1 2HE, Lancashire, UK

**Keywords:** cardiac rehabilitation, nutrition, education, systolic blood pressure, cardiovascular disease

## Abstract

This trial examined the influence of two nutrition intervention programmes on health-related and nutritional intake indices pertinent to cardiovascular disease compared to usual care in stage IV cardiac rehabilitation patients. Sixty-six patients were recruited from the Heartbeat North-West cardiac rehabilitation centre in Preston (United Kingdom). Patients were randomly assigned to Usual care, Biggest loser or Nutrition education arms. Usual care undertook their usual two exercise classes per week without nutritional support, Biggest loser underwent weekly education sessions focussing on weight loss using the themes within the British Heart Foundation ‘So You Want to Lose Weight for Good’ guidelines, and Nutrition education followed the same themes as Biggest loser but in a more focussed manner informed by patient focus groups. In total, this was a 12-week trial in which patients spent 6 weeks engaged in their allocated intervention arm, after which all participants switched to Usual care. The primary outcome was systolic blood pressure, and secondary measures of anthropometric, blood biomarker, nutritional knowledge (via the Mediterranean Diet Assessment Tool) and nutritional intake indices were measured at baseline, 6 weeks, and 12 weeks (follow-up). Intention-to-treat analyses revealed no significant alterations in the primary outcome (Usual care: baseline = 130.45 mmHg, 6 weeks = 127.83 mmHg, and follow-up = 126.35 mmHg, Biggest loser: baseline = 133.50 mmHg, 6 weeks = 123.06 mmHg, and follow-up = 135.22 mmHg, or Nutrition education: baseline = 135.23 mmHg, 6 weeks = 129.20 mmHg, and follow-up = 126.26 mmHg) between arms. However, at 6 weeks, improvements in triglycerides were significantly greater in Usual care (baseline = 1.61 mmol/L and 6 weeks = 1.43 mmol/L) group compared to Nutrition education (baseline = 1.70 mmol/L and 6 weeks = 2.21 mmol/L). Improvements in nutrition knowledge were significantly greater in Nutrition education (baseline = 8.48, 6 weeks = 9.77, and follow-up = 10.07) compared to Usual care (baseline = 7.71, 6 weeks = 8.00, and follow-up = 8.00) and Biggest loser (baseline = 7.71, 6 weeks = 8.73, and follow-up = 8.36). At 6 weeks, the Biggest loser group (baseline = 4.75 g and 6 weeks = 3.83 g) exhibited significantly greater improvements in salt intake compared to Usual care (baseline = 4.87 g and 6 weeks = 4.28 g) and Nutrition education (baseline = 19.25 g and 6 weeks = 16.78 g) in saturated fatty acids compared to Usual care (baseline = 20.26 g and 6 weeks = 21.34 g). This trial shows the effectiveness of nutritional interventions for improving nutritional knowledge and dietary practices in stage IV cardiac rehabilitation, but the mechanisms and longer-term effects of increased triglyceride levels in the Nutrition education group requires further exploration.

## 1. Introduction

Cardiovascular disease is characterised by disorders of the heart and blood vessels [1], including but not limited to coronary artery disease, stroke, peripheral artery disease, cerebrovascular disease, rheumatic heart disease, congenital heart disease and deep vein thrombosis [2]. Cardiovascular disease is recognised as the predominant cause of mortality and acknowledged as one of the most concerning chronic disease modalities [3]. Globally, it is estimated that 32% of annual fatalities are attributable to this condition, [4] and it is projected that this pathology will be the cause of more than 23 million deaths by 2030 [5]. In the United Kingdom (UK), it is estimated that 29% of all deaths occur from cardiovascular disease, equating to a death rate of 176,000 people per year [6]. The monetary effects of cardiovascular disease in the UK are considerable with the direct and indirect fiscal implications of this condition exceeding £9 billion and £19 billion, respectively [7].

Following acute myocardial infarction, patients are advised to engage in cardiac rehabilitation programs in conjunction with their routine medical treatment and subsequent check-ups [8]. In the UK, suitable cardiac patients undergo a four-phase programme of cardiac rehabilitation [8]. According to the British Heart Foundation National Audit of Cardiac Rehabilitation Quality and Outcomes Report, there are almost 100,000 individuals engaged in cardiac rehabilitation in the UK [9]. The demographic analyses showed that this comprised 71% males and 29% females and 79% British, 5% Irish/any other White background, 3% Indian, 3% Pakistan and the remaining 10% were either from other Asian, Mixed/Multiple ethnic groups, Asian/Asian British, Black/African/Caribbean/Black British groups or not-stated [9]. Cardiac rehabilitation in the UK is provided across 233 delivery programmes, of which 20 are located within the Greater Manchester, Lancashire and South Cumbria region (where this trial was undertaken) [9]. The first three aspects of the cardiac rehabilitation continuum are delivered by the National Health Service [10]. Phase I represents the in-hospital component of the rehabilitation program, phase II is characterised as the early outpatient/convalescence phase, and phase III is characterised as the outpatient/training with supervision [11]. Phase IV, which focuses on long-term maintenance of physical activity and lifestyle change, is characterised by supervised circuit-based exercise classes undertaken within the community [12]. Phase IV of cardiac rehabilitation is now well established in the UK, with an audit undertaken by the British Association for Cardiovascular Prevention & Rehabilitation (BACPR) showing that there were over 700 phase IV classes held each week across the UK to more than 15,000 participants [13].

Physical activity and exercise are recognised as health-enhancing modalities [14]. The World Health Organisation (WHO) has denoted that a lack of physical activity is among the principal risk factors for non-communicable diseases and total mortality [15]. Whilst a lack of physical activity has been shown to be responsible for more than 9% of premature fatalities globally [16], it has been shown to significantly attenuate non-communicable diseases, including cardiovascular disease [17]. Crucially, there exists compelling evidence showing that regular physical exercise is linked to diminished symptoms, decreased mortality, and reduced relapse rates among participants in cardiac rehabilitation [18,19,20]. Importantly, phase IV of cardiac rehabilitation has been shown to mediate statistically significant improvements in physical fitness, cardiometabolic and health-related quality of life indices compared to those who do not engage in this phase of the rehabilitation continuum [11,21].

In post-myocardial infarction patients, the adoption of a higher quality diet has been linked to a reduced risk of cardiovascular disease-related incidents and overall mortality [22]. As such, nutrition behaviour change, as well as revision of dietary patterns, are considered core components of cardiac rehabilitation [23]. However, according to a recent audit by the National Audit of Cardiac Rehabilitation (NACR), only 53% of cardiac rehabilitation patients have access to a nutritionist/dietitian, and in practice, this access is often limited to a single session following phase II of the rehabilitation process [24]. Notably, in patients examined 1 year following diagnosis with cardiovascular disease, it has been shown that a high proportion of patients reported poor dietary quality with only small percentages meeting the recommended consumption of basic food groups: 12.4% for vegetables, 7.8% for fruit, 8% for cereal fibre and 5.2% for trans-fat intake [25]. Importantly, our research in patients undertaking phase IV cardiac rehabilitation has shown that the majority of patients did not meet the 5-a-day recommendations for fruit and vegetables and also demonstrated notable gaps in their knowledge in areas concerning low-fat products, identifying foods that are high in added sugar, and connecting the association between diet and effective disease management [26]. It has been proposed with the latter stages of cardiac rehabilitation being associated predominantly with exercise-based behaviour modification that future behavioural interventions should include both physical activity and nutritional modification for optimum results [25].

### 1.1. Rationale

Previous analyses have shown that stage IV is effective in mediating improvements in physical fitness, cardiometabolic and health-related quality of life [12,21], although recent evidence shows that nutritional knowledge is lacking in this patient group [26] and remains an overlooked component of disease management [24]. The delivery of effective nutritional interventions appears to represent an ideal solution to improve disease management in stage IV cardiac rehabilitation patients. The British Heart Foundation has developed ‘so you want to lose weight for good’ healthy eating guidelines [27] that are adopted in cardiac rehabilitation [26] and referred to as Biggest loser. In addition, our research [26] of stage IV cardiac rehabilitation patients has shown that dietary guidelines are overly restrictive in that they predominantly advise what should not be consumed rather than being inclusive of what they should eat. Based on this information, a new more nutritionally inclusive intervention has been developed termed Nutrition education. 

There are currently no published randomised intervention analyses concerning the efficacy of nutrition interventions undertaken within stage IV of the cardiac rehabilitation continuum. Therefore, given the well-established association between dietary intake and cardiovascular disease management/aetiology [22], allied to the lack of nutrition knowledge in this group [26], randomised interventions examining the effects of the Biggest loser and Nutrition education interventions appear to be strongly warranted. 

### 1.2. Aims 

The purpose of this trial is to undertake a three-arm randomised control trial examining the effects of two nutrition intervention programmes (Biggest loser and Nutrition education) compared to Usual care in stage IV cardiac rehabilitation patients. The primary objective of this trial is to examine the influence of the aforementioned programmes on systolic blood pressure relative to Usual care. The secondary objectives are to determine whether the nutrition interventions impact other anthropometric, blood lipids, nutritional knowledge and nutritional intake indices outcomes pertinent to cardiovascular disease.

### 1.3. Hypotheses

In relation to the primary outcome, the Biggest loser and Nutrition education trial arms will mediate more substantial reductions in systolic blood pressure levels compared to Usual care. Furthermore, for the secondary outcomes of blood pressure, anthropometric indices, and nutritional intake, we anticipate that these will also improve to a greater extent as a function of the Biggest loser and Nutrition education interventions compared to Usual care.

## 2. Materials and Methods

### 2.1. Study Design and Setting

**Figure 1:**
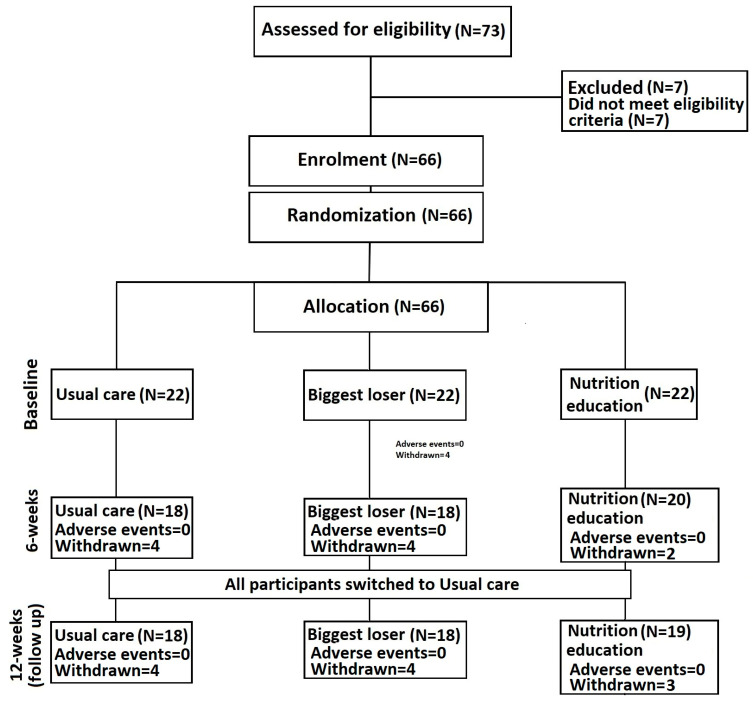
Consort diagram showing participant flow throughout the study.

This examination comprises a parallel, randomised controlled trial spanning 12 weeks (Figure 1). The intervention period of 12 weeks, along with subsequent follow-up, adhered to the guidelines established in [28], and the protocol was formulated based on the revised recommendations for reporting parallel group randomised controlled trials [29]. The University of Central Lancashire conducted the current investigation at the Heartbeat North-West cardiac rehabilitation centre located in Preston within the county of Lancashire, United Kingdom.

Following the eligibility screening and enrolment process, participants underwent individual randomisation through a computer program (Random Allocation Software) into a 6-week period of either Usual care, Biggest loser or Nutrition education trial arms. After the initial 6-week intervention period, all participants then switched to Usual care for 6 weeks. A follow-up time point was adopted to examine whether there were any persistent or enhanced effects following cessation of each intervention arm. Primary and secondary outcome variables, as described in detail below, were assessed at baseline, 6-weeks and at 12-weeks follow up. In agreement with previous trials of cardiovascular disease management, the primary outcome measure was the between-group change in systolic blood pressure [30]. Secondary outcome measures were between-group differences in anthropometric, diastolic blood pressure, blood lipids, nutrition knowledge and nutritional intake.

### 2.2. Inclusion Criteria

Inclusion criteria included the capacity to give written informed consent, engaged in phase IV cardiac rehabilitation and attending at least two structured exercise classes per week delivered by the Heartbeat North-West rehabilitation service.

### 2.3. Exclusion Criteria

The exclusion criteria included any recent change in condition, dietary intake or medication that would interfere with the trial findings, having specific dietary needs and engagement in another randomised controlled trial.

### 2.4. Sample Size

Calculations for sample size were executed for the primary outcome variable, i.e., the between-group difference in systolic blood pressure. The analysis indicated that a total sample size of 66 was needed to achieve 80% power in detecting a clinically significant change of 5 mmHg between groups [31], assuming a projected standard deviation of 5.5 mmHg in each group [32], while factoring in an anticipated 20% loss to follow-up rate.

### 2.5. Participants and Recruitment

This study was conducted with patients attending the Heartbeat North-West cardiac rehabilitation centre located in Preston. Recruiting materials were placed in the cardiac rehabilitation centre using public patient bulletin boards, and participants were enrolled during April–June 2018. Individuals expressing interest in participation were provided with the chance to reach out to the research team for additional details about the study and to address any questions related to participation. Written informed consent was acquired, and all participants were instructed to continue their regular medication regimen. Additionally, participants in the Usual care group were asked to maintain their current dietary patterns until the completion of the final ‘follow-up’ data collection session.

### 2.6. Ethical Approval and Trial Registration

Approval for the study was granted by an institutional ethical review board (STEMH 634), and all participants submitted written informed consent before participating, adhering to the principles stated in the Declaration of Helsinki. The trial was preregistered on clinicaltrials.gov (NCT05198024).

### 2.7. Intervention

#### 2.7.1. Usual Care

The Usual care group involved patients who attended the standard stage IV cardiac rehabilitation program offered within the Heartbeat centre in Preston. Patients were asked to continue with their habitual regimen of attending the two weekly structured exercise classes delivered by a level 4 BACPR accredited practitioner and also to maintain their habitual dietary intake during the study period. 

#### 2.7.2. Biggest Loser

In addition to participating in the structured exercise classes attended by the Usual care group, individuals assigned to the Biggest Loser arm also underwent a six-week nutrition intervention program. The program, conducted by a nutritionist for one hour weekly at the Heartbeat North-West centre in Preston, outside of the patients’ regular exercise class schedule, aimed to enhance awareness of the risks associated with a high body mass index (BMI) and waist circumference measurement. Each weekly session followed a designated theme aligned with the British Heart Foundation’s ‘So You Want to Lose Weight for Good’ healthy eating guidelines [27], covering: (1) increased consumption of fruits and vegetables, (2) elevated intake of fish, (3) reduction in saturated fat intake, (4) decrease in salt consumption, (5) adherence to safe alcohol limits, and (6) meal planning and goal setting.

#### 2.7.3. Nutrition Education

In addition to participating in the structured exercise classes attended by the Usual care group, individuals assigned to the Nutrition education arm also underwent a six-week nutrition intervention program. This program was conducted at the Heartbeat North-West centre in Preston, outside of patients’ regular exercise class times, by the same nutritionist. The Nutrition education group covered the same weekly topics as the Biggest loser trial arm, but the delivery materials and content were shaped by insights gained from our patient involvement focus groups. Additionally, our patient involvement focus groups, in response to previously observed deficiencies in nutritional behaviour and knowledge [26], highlighted three main themes: barriers, confusion, and inclusion. Barriers to attending nutrition education included reluctance to go on a diet, not needing to lose weight, time constraints, and concerns about the trustworthiness of the person delivering the nutrition education session. Confusion centred around how to meet low salt, sugar, and fat targets and determine appropriate portion sizes. Inclusion was driven by patients’ desire to understand what they could and should eat, rather than just focusing on what to omit, along with a request for simple recipes and meals for one.

In the first week, patients received a basic ring binder, plastic wallets, and dividers. Throughout the intervention, they were given printouts of lecture slides, portion information for each food group, recipes, and additional materials pertinent to the weekly topic. This comprehensive self-help guide aimed to offer patients a thorough resource for heart-healthy eating. Each of the six sessions focused on what should be included for heart health, which was supported by research informing these recommendations. Lecture slides were meticulously crafted to present colourful and informative content, with active participation encouraged throughout each session. Heart-healthy handouts utilised literature from the British Heart Foundation, covering topics such as portion sizes and simple recipes.

### 2.8. Data Collection

#### 2.8.1. Anthropometric Measurements

Anthropometric measures were undertaken by a level 4 BACPR accredited practitioner at the same times for each test prior to the commencement of patients’ scheduled exercise class. Measures of mass (kg) and stature (m) (without shoes) were utilised to calculate the body mass index (BMI) (kg/m^2^). Stature was gauged using a stadiometer (Seca, Hamburg, Germany), while mass was measured using weighing scales (Seca 875, Hamburg, Germany). Waist circumference was ultimately measured at the midpoint between the inferior margin of the last rib and the iliac crest, and hip circumference was taken around the pelvis at the point of maximum buttock protrusion, without compressing the soft tissues [33], enabling the quantification of the waist-to-hip ratio.

#### 2.8.2. Haematological Testing

Capillary blood samples were obtained through finger-prick procedures, using a disposable lancet after cleaning using an ethanol wipe. Three handheld analysers (Multi-careIn, Multicare Medical, Arezzo, Italy) were employed to measure capillary triglyceride, total cholesterol, and glucose levels (mmol/L). From these results, the triglyceride glucose index were calculated as the natural logarithm of the product of plasma glucose and triglycerides, divided by two [34].

#### 2.8.3. Blood Pressure 

Measurements of blood pressure (mmHg) were conducted with individuals in an upright seated position. Peripheral measurements of systolic and diastolic blood pressure were obtained using a non-invasive blood pressure monitor (OMRON M2, Kyoto, Japan), following the guidelines outlined by the European Society of Hypertension [35]. Three readings were taken, each spaced apart by 1 min [36], and the mean of the last 2 readings was utilised for analysis.

#### 2.8.4. Questionnaires

All questionnaires were presented in paper format. To examine dietary practices, the 14-iten Mediterranean Diet Assessment Tool was utilised to quantify nutritional knowledge and competency [37]. This questionnaire has been shown to be associated with a range of cardiovascular disease indices [38], and a higher score is associated with lower risk of cardiovascular disease and all-cause mortality [39]. Furthermore, to explore changes in dietary practices between trial arms, 4-day diet diaries were completed for the days prior each assessment time point [40]. Diet diaries were analysed using WinDiets Nutritional Analysis Software Suite (Robert Gordon University, Aberdeen, UK), allowing daily energy intake, fat, saturated fatty acids, protein, carbohydrate, free sugars, fibre, alcohol, vitamin A, thiamine, riboflavin, niacin, vitamin B6, vitamin B12, folate, vitamin C, calcium, salt, iron, zinc and selenium to be examined. Training in relation to the diet diaries was provided by the nutritionist to all trial arms, and the diet diaries themselves included images and text showing examples of portion sizes [41]. 

### 2.9. Statistical Analyses

Descriptive statistics, including means and standard deviations, were provided for each continuous outcome measure, while categorical outcomes are presented as total number. Comparisons between trial arms at baseline were undertaken using between subjects using linear mixed effects models for continuous outcomes with the trial arm modelled as a fixed factor and random intercepts by participants. Comparison of the number of males/females between trial arms was undertaken using two-way Pearson chi-square tests of independence.

Furthermore, in order to contrast the magnitude of the changes in primary and secondary outcomes at both 6-weeks and 12-weeks follow-up between the trial arms, linear mixed effects models were employed, with group modelled as a fixed factor and random intercepts by participants adopted, adjusting for covariate baseline scores, age and gender [42]. For linear mixed models, the adjusted mean difference (*b*), t-value, and 95% confidence intervals of the difference are reported. These analyses were conducted on an intention-to-treat basis, employing the restricted maximum-likelihood method [14].

Two-way Pearson chi-square tests of independence were employed for bivariate cross-tabulation comparisons among the three trial groups, assessing the number lost to follow-up and the occurrence of adverse outcomes in each group. Chi-square probability values were computed using Monte Carlo simulation. All statistical analyses for significance were performed using SPSS v27 (IBM, SPSS). A significance level of *p* < 0.05 was considered for all analyses. For conciseness and clarity, only variables demonstrating statistical significance attributable to the intervention are presented in the Results section.

## 3. Results

### 3.1. Baseline Characteristics

Comparisons between continuous outcomes between trial arms at baseline were non-significant (*p* = 0.10–0.99) with the exception of stature, which was shown to be greater in the Usual care group compared to Nutrition education (*b* = 0.03, (95% CI = 0.008–0.06), t = 2.69, *p* = 0.01). The chi-squared test contrasting the number of males/females between trial arms was non-significant (*X*^2^ _(2)_ = 4.03, *p* = 0.133) (Table 1).

### 3.2. Loss to Follow-Up and Adverse Events

At 6 weeks, the loss to follow-up in each group for Usual care (N = 4), Biggest loser, (N = 4) and Nutrition education (N = 2) was similar to that at 12 weeks: Usual care (N = 4), Biggest loser (N = 4) and Nutrition education (N = 3). The total number of adverse effects were Usual care (N = 0), Biggest loser (N = 0) and Nutrition education (N = 0) (Figure 1). Chi-square tests for loss to follow-up and adverse events were non-significant (*p* = 0.689–1.00), indicating that there were no statistically significant differences between trial arms in either loss to follow up or adverse events (Figure 1).

### 3.3. Anthropometric Measurements

No significant differences in anthropometrics were evident between groups (Table 2 and Table 3).

### 3.4. Haematological

Improvements in triglycerides at 6 weeks were significantly greater in the Usual care (*b* = 0.33, (95% CI = 0.03–0.61), *p* = 0.025) group compared to Nutrition education (Table 2 and Table 3).

### 3.5. Blood Pressure 

No significant differences in blood pressure were evident between groups (Table 2 and Table 3).

### 3.6. Nutrition Knowledge

Improvements in Mediterranean Diet Assessment Tool scores were significantly greater at 6 weeks (*b* = 0.62, (95% CI = 0.10–1.13), t = 2.45, *p* = 0.021) and 12 weeks (*b* = 0.91, (95% CI = 0.29–1.53), t = 3.02, *p* = 0.005) in the Nutrition education group compared to Usual care. Furthermore, improvements in Mediterranean Diet Assessment Tool scores at 6 weeks were significantly greater (*b* = 1.79, (95% CI = 0.62–2.97), t = 3.15, *p* = 0.004) in the Nutrition education group compared to Biggest loser (Table 2 and Table 3).

### 3.7. Dietary Practices

In addition, the Biggest loser group exhibited significantly greater improvements in salt intake at 6 weeks (*b* = 0.83, (95% CI = 0.10–1.55), t = 2.32, *p* = 0.027) compared to Usual care (Table 4 and Table 5). Furthermore, the improvements in saturated fatty acids (*b* = 2.47, (95% CI = 0.51–4.44), t = 2.58, *p* = 0.015) were significantly greater at 6 weeks in the Nutrition education group compared to Usual care (Table 4 and Table 5). Finally, improvements in free sugar intake at 12 weeks were significantly greater (*b* = 7.12, (95% CI = 2.60–11.63), t = 3.22, *p* = 0.003) in the Nutrition education group compared to Usual care (Table 4 and Table 5).

## 4. Discussion

The aim of the current study was to investigate the influence of two nutrition intervention programmes on systolic blood pressure, other health-related and nutritional intake compared to Usual care in patients undergoing stage IV cardiac rehabilitation. Owing to the well-established association between dietary intake and cardiovascular disease management/aetiology allied to the lack of nutrition knowledge in this group, a nutrition intervention delivered within stage IV cardiac rehabilitation appears to be warranted. To date, this represents the first investigation to explore the effects of a nutritional intervention using a randomised controlled trial in stage IV cardiac rehabilitation. The primary objective was to assess the impact of nutritional intervention on systolic blood pressure, while the secondary goal(s) were to investigate the effects of nutrition intervention on various anthropometric, blood lipid, nutritional knowledge, and nutritional intake indices pertinent to cardiovascular disease.

Regarding the primary outcome, the results from the present study do not align with our hypothesis, as there were no notable reductions in systolic blood pressure in either of the nutrition intervention trial arms compared to Usual care. It is not within the scope of the biological measures obtained during this trial to elucidate the mechanism(s) responsible for the lack of improvement in systolic blood pressure. However, taking into account the study durations adopted in previous trials exploring the effects of drug therapy in the treatment of hypertension [43], it can be speculated that the study duration was not sufficient to observe improvements in the primary outcome. Regardless, the observations from the current investigation indicate that nutritional interventions, as specifically adopted in the current investigation, do not appear to be effective in mediating improvements in systolic blood pressure in patients engaged in stage IV cardiac rehabilitation following 6-week intervention and follow-up periods. As the adoption of a higher quality diet has been linked to a reduced risk of cardiovascular disease-related incidents and overall mortality [22], it is recommended that future randomised investigations consider longer nutrition education intervention durations.

The results from this trial did, however, reveal significant observations in secondary haematological indices. In opposition to our hypothesis, triglyceride improvements were shown to be significantly greater in Usual care compared to Nutrition education. This observation is an interesting one from an efficacy standpoint. A long-standing link exists between elevated triglyceride levels and cardiovascular disease [44], and although triglycerides themselves are not directly atherogenic, they epitomise an important biomarker of cardiovascular disease aetiology and progression, owing to their association with atherogenic remnant particles and apo CIII [45]. It is again not within the scope of the biological measurements examined in this trial to accurately determine the mechanisms responsible for this finding. The current investigation does nonetheless suggest that neither nutrition intervention was more effective than Usual care in terms of in facilitating improved haematological parameters; and notably, the Nutrition education group fared less favourably in relation to alterations in triglycerides.

However, although no significant improvements in the primary outcome or key anthropometric indices, i.e., body mass, waist circumference or waist-to-hip ratio, were observed, this trial showed that the Nutrition education intervention mediated greater improvements in nutrition knowledge compared to Usual care and Biggest loser interventions. This is an important observation firstly as the improvements were maintained at 12 weeks follow-up, but also as higher Mediterranean diet scores have been associated with a reduced risk of mortality due to cardiovascular disease [38,39]. Importantly, given the nature of the intervention groups, this trial also showed improvements in aspects of dietary intake pertinent to cardiovascular disease. Specifically, the Biggest loser intervention produced significantly greater improvements in salt intake, and the Nutrition education produced statistically significant improvements in saturated fatty acid and free sugar intake. These observations appear to be promising given the recently published Dietary Guidelines for Americans [46], emphasising that individuals limit the intake of saturated fats, salt and sugar. However, such observations in relation to nutritional intake do not provide any insight into the lack of improvement in primary and haematological outcomes and appear to be contradictory given the increased triglyceride levels in the Nutrition education group. Regardless, the observations from the current trial suggest that the Nutrition education intervention was able to mediate improvements in nutritional knowledge and that both intervention modalities adopted in the current trial are able to successfully improve dietary intake in stage IV cardiac rehabilitation patients and may therefore be important for future disease management.

Overall, the current trial demonstrated a high retention rate in both intervention groups without evidence of any adverse effects, and importantly, it showed that both intervention groups mediated improvements in pertinent nutritional indices. This indicates that nutritional interventions appear to be safe and tolerable modalities within stage IV cardiac rehabilitation. Although, this trial demonstrated positive outcomes in dietary practices and nutrition knowledge, it was not a part of this investigation to examine the fiscal implications of delivering nutrition interventions in stage IV cardiac rehabilitation. It was therefore beyond the scope of this trial to undertake a cost-effectiveness analysis, but future work should seek to explore the economic value of nutrition education interventions to provide insights for the allocation of healthcare resources and implementation at a wider level. 

As with all experimental research, this trial is not without limitations. Firstly, that exercise intensity undertaken as part of patients twice-weekly exercise classes was not standardised or quantified as part of the current trial may serve as a potential drawback. Previous analyses have shown that exercise intensity plays an important role in the aetiology and management of cardiovascular disease and its risk factors [47]. Therefore, examination of exercise intensity throughout the intervention period could have offered additional information regarding the mechanisms responsible for some of the observations in this investigation, such as the increased triglycerides that were evident in the Nutrition education trial arm. Therefore, despite the inherent challenges associated with the accurate quantification of exercise intensity [14], future analyses examining the effects of nutritional interventions in stage IV cardiac rehabilitation should nonetheless seek to examine the intensity of the exercise undertaken during the intervention period. The fact that primary and secondary blood pressure indices were undertaken in an acute manner in a laboratory environment may also represent a limitation to the current trial. Although more logistically and fiscally challenging, twenty-four ambulatory blood pressure could be more advantageous in establishing alterations in blood pressure following nutritional interventions, as it provides a more accurate depiction of systemic blood pressure throughout an entire 24-hour time period and reduces the likelihood of white-coat hypertensive readings [48]. Additionally, the randomisation of participants into their respective trial arms without accounting for their prior levels of physical activity, medication, disease activity, or other experimental factors could be viewed as a potential limitation. Although comparative analyses at baseline revealed mostly small and non-significant differences (except for stature) between trial arms, future analyses could consider implementing a stratified random sampling approach. This approach could be beneficial when investigating the effects of nutritional intervention on indices relevant to the aetiology and management of cardiovascular disease.

## 5. Conclusions

The aim of the current study was to investigate, using a randomised controlled trial, the influence of two nutrition intervention programmes on systolic blood pressure and other health-related and nutritional intake indices pertinent to cardiovascular disease in patients undergoing stage IV cardiac rehabilitation. This trial notably showed no significant improvements in systolic blood pressure, but significant improvements in triglycerides were noted in the Usual care group compared to Nutrition education. However, the Nutrition education group mediated statistical improvements in nutrition knowledge, and both Nutrition education and Usual care trial arms led to significant improvements in the dietary intake of saturated fatty acids, salt and sugar. This study shows that nutritional interventions can improve nutritional knowledge and dietary practices in stage IV cardiac rehabilitation patients, but the mechanisms and longer-term effects of increased triglyceride levels in the Nutrition education group requires further exploration. Future randomised investigations should consider longer nutrition education intervention durations.

## Figures and Tables

**Table 1:** Baseline characteristics (mean ± standard deviation (*SD*)).

	Usual Care		Biggest Loser		Nutrition Education	
	Mean	*SD*	Mean	*SD*	Mean	*SD*
Age (years)	68.80	8.69	68.05	9.29	69.14	7.85
Sex (Male/Female)	16/4		17/5		12/10	
Stature (m)	1.72	0.08	1.70	0.10	1.65	0.09
Body mass (kg)	86.21	14.02	88.28	23.00	83.49	24.08
Body mass index (kg/m^2^)	29.41	5.78	30.51	8.29	30.57	7.72

**Table 2:** Mean ± standard deviation (*SD*) anthropometric, haematological, blood pressure and nutritional knowledge outcomes for each trial arm.

	Usual Care						Biggest Loser						Nutrition Education					
	Baseline		6-Weeks		12-Weeks		Baseline		6-Weeks		12-Weeks		Baseline		6-Weeks		12-Weeks	
	Mean	*SD*	Mean	*SD*	Mean	*SD*	Mean	*SD*	Mean	*SD*	Mean	*SD*	Mean	*SD*	Mean	*SD*	Mean	*SD*
Body mass (kg)	86.21	14.02	83.23	8.23	82.69	8.70	88.28	23.00	85.54	24.88	87.04	24.30	83.49	24.08	82.25	25.46	82.05	26.37
Body mass index (kg/m^2^)	29.41	5.78	28.04	3.84	27.96	4.00	30.51	8.29	30.12	9.22	30.41	8.84	30.57	7.72	29.82	7.99	29.73	8.25
Waist circumference (cm)	103.35	10.92	99.94	9.05	100.88	7.48	104.86	15.29	100.88	14.79	103.78	14.55	103.23	19.65	101.00	19.15	99.79	20.51
Waist to hip ratio	0.96	0.06	0.94	0.06	0.95	0.05	0.97	0.08	0.95	0.09	0.98	0.10	0.93	0.08	0.95	0.07	0.93	0.09
Systolic blood pressure (mm/Hg)	130.45	11.10	127.83	18.50	126.35	15.58	133.50	21.55	123.06	18.99	135.22	23.36	135.23	20.16	129.20	28.82	126.26	18.98
Diastolic blood pressure (mm/Hg)	74.40	9.72	72.17	7.58	72.12	11.70	78.27	10.28	72.65	10.78	78.22	12.50	72.05	12.00	70.30	11.91	71.84	13.42
Total cholesterol (mmol/L)	4.20	0.71	4.50	0.70	4.49	0.61	4.39	0.95	4.67	0.95	4.28	1.01	4.47	0.70	5.03	0.69	4.59	0.45
Glucose (mmol/L)	7.27	2.64	6.91	2.31	6.71	2.37	7.55	1.93	7.37	2.79	7.23	1.88	6.83	1.64	7.51	3.52	7.10	2.39
Triglycerides (mmol/L)	1.61	1.10	1.43	0.67	1.54	1.08	1.90	1.20	1.86	1.23	1.92	0.96	1.70	0.78	2.21	0.99	1.84	0.74
Triglyceride glucose index	4.80	0.36	4.72	0.27	4.75	0.37	4.91	0.37	4.89	0.36	4.93	0.32	4.84	0.28	5.00	0.32	4.90	0.27
Mediterranean Diet score	7.71	2.37	8.00	1.87	8.00	2.39	7.71	2.35	8.73	2.49	8.36	2.46	8.48	2.20	9.77	1.59	10.07	1.73

**Table 3:** Mean change scores for anthropometric, haematological, blood pressure and nutritional knowledge outcomes for each trial arm.

	Usual Care		Biggest Loser		Nutrition Education		
	Baseline–6 Weeks	Baseline–12 Weeks	Baseline–6 Weeks	Baseline–12 Weeks	Baseline–6 Weeks	Baseline–12 Weeks	
Body mass (kg)	−2.98	−3.52	−2.74	−1.24	−1.24	−1.44	
Body mass index (kg/m^2^)	−1.37	−1.45	−0.39	−0.1	−0.75	−0.84	
Waist circumference (cm)	−3.41	−2.47	−3.98	−1.08	−2.23	−3.44	
Waist to hip ratio	−0.02	−0.01	−0.02	0.01	0.02	0.00	
Systolic blood pressure (mm/Hg)	−2.62	−4.1	−10.44	1.72	−6.03	−8.97	
Diastolic blood pressure (mm/Hg)	−2.23	−2.28	−5.62	−0.05	−1.75	−0.21	
Total cholesterol (mmol/L)	0.30	0.29	0.28	−0.11	0.56	0.12	
Glucose (mmol/L)	−0.36	−0.56	−0.18	−0.32	0.68	0.27	
Triglycerides (mmol/L)	−0.18	−0.07	−0.04	0.02	0.51	0.14	A
Triglyceride glucose index	−0.08	−0.05	−0.02	0.02	0.16	0.06	
Mediterranean Diet score	0.29	0.29	1.02	0.65	1.29	1.59	B, C, D

Notes: A = significantly greater change scores from baseline to 6 weeks in Usual care vs. Nutrition education; B = significantly greater change scores from baseline to 6 weeks in Nutrition education vs. Usual care; C = significantly greater change scores from baseline to 12 weeks in Nutrition education vs. Usual care; D = significantly greater change scores from baseline to 6 weeks in Nutrition education vs. Biggest loser.

**Table 4:** Mean ± standard deviation (*SD*) daily dietary outcomes for each trial arm.

	Usual Care						Biggest Loser						Nutrition Education					
	Baseline		6 Weeks		12 Weeks		Baseline		6 Weeks		12 Weeks		Baseline		6 Weeks		12 Weeks	
	Mean	*SD*	Mean	*SD*	Mean	*SD*	Mean	*SD*	Mean	*SD*	Mean	*SD*	Mean	*SD*	Mean	*SD*	Mean	*SD*
Energy intake (Kcal)	1761.69	420.88	1656.06	373.09	1714.73	512.96	1676.79	441.06	1596.94	585.22	1603.10	465.56	1516.00	387.23	1362.83	285.15	1573.00	412.81
Fat (g)	62.21	19.83	61.52	16.25	68.76	29.18	58.85	25.80	56.49	25.57	56.45	19.02	56.56	19.26	52.92	13.98	59.36	17.07
Saturated fatty acids (g)	20.26	5.46	21.34	6.34	23.37	10.33	22.52	9.62	19.72	8.64	19.39	7.53	19.25	7.58	16.78	3.82	20.75	6.16
Protein (g)	82.48	19.16	74.16	14.15	81.21	19.97	72.19	14.97	71.22	15.02	69.07	15.91	73.19	16.55	75.94	19.75	75.68	18.62
Carbohydrate (g)	219.33	77.50	193.55	60.95	192.55	63.03	205.65	61.63	189.24	87.81	195.66	93.90	175.57	49.52	142.89	57.48	183.83	58.45
Free sugars (g)	33.61	20.91	30.41	18.97	36.14	19.25	40.95	18.85	33.78	18.96	27.86	16.01	29.16	15.58	23.13	14.06	23.14	12.85
Fibre (g)	19.51	5.63	18.45	5.25	16.37	7.83	16.44	5.55	16.16	7.36	17.53	5.75	15.27	4.73	16.78	4.65	17.24	3.61
Alcohol (mL)	6.88	9.69	8.90	12.78	7.56	10.80	12.52	14.74	10.74	9.91	14.50	18.24	7.97	9.14	4.47	4.83	5.18	11.14
Vitamin A (ug)	575.19	270.44	578.94	462.32	515.47	206.97	513.58	243.24	498.38	301.00	548.80	321.36	469.23	231.40	567.17	331.97	490.40	243.01
Thiamine (mg)	1.47	0.34	12.50	44.97	1.43	0.53	1.56	0.64	1.53	0.63	1.50	0.58	1.47	0.49	1.36	0.42	1.43	0.27
Riboflavin (mg)	1.81	0.61	1.79	0.69	1.70	0.58	1.74	0.81	1.72	0.81	1.59	0.83	1.52	0.38	1.40	0.37	1.47	0.39
Niacin (mg)	32.96	6.52	31.29	8.01	31.32	9.62	32.23	9.06	33.67	10.09	29.83	7.02	29.96	7.73	30.33	9.95	29.41	10.07
Vitamin B6 (mg)	1.54	0.29	1.70	0.52	1.55	0.46	1.59	0.64	1.61	0.79	1.59	0.39	1.41	0.40	1.38	0.39	1.37	0.32
Vitamin B12 (mg)	4.41	1.69	4.93	3.24	4.45	1.55	4.04	1.84	4.60	2.73	4.08	2.38	4.51	1.94	5.09	2.34	4.09	2.05
Folate (ug)	232.44	51.80	237.47	86.14	227.80	85.02	235.84	111.35	221.56	88.43	228.10	88.99	208.82	66.84	226.58	43.25	216.00	32.02
Vitamin C (mg)	83.99	65.58	75.75	34.90	70.12	39.72	78.53	50.85	78.14	46.89	85.63	38.71	81.17	58.11	86.35	19.01	88.61	43.38
Calcium (mg)	942.81	688.96	764.47	188.83	728.87	148.33	859.47	314.02	717.75	266.18	742.70	303.60	785.91	203.75	676.58	185.86	743.47	255.10
Salt (g)	4.87	1.93	4.28	1.20	4.53	1.31	4.75	1.23	3.83	1.07	4.78	3.72	4.34	1.39	3.94	1.23	4.63	1.80
Iron (mg)	12.25	6.66	9.68	3.31	10.10	3.83	10.46	5.14	10.64	6.33	10.75	5.06	9.02	2.53	8.44	1.63	8.61	1.64
Zinc (mg)	9.19	2.03	7.89	2.06	8.18	2.39	7.91	1.76	7.81	2.01	7.82	2.17	10.26	10.46	8.15	1.77	7.67	1.62
Selenium (mg)	58.50	22.63	48.59	19.21	49.60	18.26	41.89	12.88	48.56	28.20	43.30	13.65	49.95	14.92	55.50	19.36	48.20	20.51

**Table 5:** Mean change scores for daily dietary outcomes for each trial arm.

	Usual Care		Biggest Loser		Nutrition Education		
	Baseline–6 Weeks	Baseline–12 Weeks	Baseline–6 Weeks	Baseline–12 Weeks	Baseline–6 Weeks	Baseline–12 Weeks	
Energy intake (Kcal)	105.63	46.96	79.85	73.69	153.17	−57.00	
Fat (g)	0.69	−6.55	2.36	2.40	3.64	−2.80	
Saturated fatty acids (g)	−1.08	−3.11	2.80	3.13	2.47	−1.50	B
Protein (g)	8.32	1.27	0.97	3.12	−2.75	−2.49	
Carbohydrate (g)	25.78	26.78	16.41	9.99	32.68	−8.26	
Free sugars (g)	3.20	−2.53	7.17	13.09	6.03	6.02	B
Fibre (g)	1.06	3.14	0.28	−1.09	−1.51	−1.97	
Alcohol (mL)	−2.02	−0.68	1.78	−1.98	3.50	2.79	
Vitamin A (ug)	−3.75	59.72	15.20	−35.22	−97.94	−21.17	
Thiamine (mg)	−11.03	0.04	0.03	0.06	0.11	0.04	
Riboflavin (mg)	0.02	0.11	0.02	0.15	0.12	0.05	
Niacin (mg)	1.67	1.64	−1.44	2.40	−0.37	0.55	
Vitamin B6 (mg)	−0.16	−0.01	−0.02	0.00	0.03	0.04	
Vitamin B12 (mg)	−0.52	−0.04	−0.56	−0.04	−0.58	0.42	
Folate (ug)	−5.03	4.64	14.28	7.74	−17.76	−7.18	
Vitamin C (mg)	8.24	13.87	0.39	−7.10	−5.18	−7.44	
Calcium (mg)	178.34	213.94	141.72	116.77	109.33	42.44	
Salt (g)	0.59	0.34	0.92	−0.03	0.40	−0.29	E
Iron (mg)	2.57	2.15	−0.18	−0.29	0.58	0.41	
Zinc (mg)	1.30	1.01	0.10	0.09	2.11	2.59	
Selenium (mg)	9.91	8.90	−6.67	−1.41	−5.55	1.75	

Notes: B = significantly greater change scores from baseline to 6 weeks in Nutrition education vs. Usual care; E = significantly greater change scores from baseline to 6 weeks in Biggest loser vs. Usual care.

## Data Availability

Data available on request.

## References

[1] (2017). WHO Cardiovascular Diseases (CVD). https://www.who.int/news-room/fact-sheets/detail/cardiovascular-diseases-(cvds).

[2] Vaduganathan M., Mensah G.A., Turco J.V., Fuster V., Roth G.A. (2022). The global burden of cardiovascular diseases and risk: A compass for future health. J. Am. Coll. Cardiol..

[3] Amini M., Zayeri F., Salehi M. (2021). Trend analysis of cardiovascular disease mortality, incidence, and mortality-to-incidence ratio: Results from global burden of disease study 2017. BMC Public Health.

[4] Moraga P., GBD Causes of Death Collaborators (2017). Global, regional, and national age-sex specific mortality for 264 causes of death, 1980-2016: A systematic analysis for the Global Burden of Disease Study 2016. Lancet.

[5] Ruan Y., Guo Y., Zheng Y., Huang Z., Sun S., Kowal P., Wu F. (2018). Cardiovascular disease (CVD) and associated risk factors among older adults in six low-and middle-income countries: Results from SAGE Wave 1. BMC Public Health.

[6] British Heart Foundation (2023). Factsheet, U.K. British Heart Foundation. https://www.bhf.org.uk/-/media/files/for-professionals/research/heart-statistics/bhf-cvd-statistics-uk-factsheet.pdf.

[7] British Heart Foundation (2017). Analysis of European Cardiovascular Disease Statistics 2017, EHN. www.ehnheart.org/cvd-statistics/cvd-statistics-2017.html.

[8] British Heart Foundation (2020). The National Audit of Cardiac Rehabilitation Quality and Outcomes Report 2020. https://www.bhf.org.uk/-/media/images/information-support/publications/hcp/nacr_quality_and_outcomes_report_2020.pdf?rev=b2d2789a242a452ea43b128e8f02c11d.

[9] Woolf-May K., Bird S. (2005). Physical activity levels during phase IV cardiac rehabilitation in a group of male myocardial infarction patients. Br. J. Sports Med..

[10] Sniehotta F.F., Gorski C., Araújo-Soares V. (2020). Adoption of community-based cardiac rehabilitation programs and physical activity following phase III cardiac rehabilitation in Scotland: A prospective and predictive study. Psychol. Health.

[11] Noites A., Freitas C.P., Pinto J., Melo C., Vieira A., Albuquerque A., Bastos J.M. (2017). Effects of a phase IV home-based cardiac rehabilitation program on cardiorespiratory fitness and physical activity. Heart Lung Circ..

[12] Atkins S., Holland S., Crossley D., Taylor P.J., Sinclair J. (2017). Assessment of the effectiveness of a phase IV cardiac rehabilitation programme. Lancet.

[13] Thow M., Hinton S., Rafferty D. A survey of phase IV cardiac rehabilitation provision in the, U.K.. Proceedings of the BACR Exercise Professionals Conference.

[14] Sinclair J., Ageely H., Mahfouz M.S., Hummadi A.A., Darraj H., Solan Y., Bottoms L. (2023). Effects of a Home-Based Physical Activity Program on Blood Biomarkers and Health-Related Quality of Life Indices in Saudi Arabian Type-2 Diabetes Mellitus Patients: A Randomized Controlled Trial. Life.

[15] World Health Organization (WHO) (2010). Global Recommendations on Physical Activity for Health.

[16] Lee I.M., Shiroma E.J., Lobelo F., Puska P., Blair S.N., Katzmarzyk P.T. (2012). Effect of physical inactivity on major non-communicable diseases worldwide: An analysis of burden of disease and life expectancy. Lancet.

[17] Warburton D.E., Nicol C.W., Bredin S.S. (2006). Health benefits of physical activity: The evidence. Can. Med. Assoc. J..

[18] Myers J., Prakash M., Froelicher V., Do D., Partington S., Atwood J.E. (2004). Exercise capacity and mortality among men referred for exercise testing. N. Engl. J. Med..

[19] O’Connor G.T., Buring J.E., Yusuf S., Goldhaber S.Z., Olmstead E.M., Paffenbarger R.S., Hennekens C.H. (1989). An overview of randomized trials of rehabilitation with exercise after myocardial infarction. Circulation.

[20] Oldridge N.B., Guyatt G.H., Fischer M.E., Rimm A.A. (1988). Cardiac rehabilitation after myocardial infarction: Combined experience of randomized clinical trials. JAMA.

[21] Willmer K., Waite M., Nurse C.R., Willmer K.A. (2009). Long-term benefits of cardiac rehabilitation: A five-year follow-up of community-based phase 4 programmes. Br. J. Cardiol..

[22] Casas R., Castro-Barquero S., Estruch R., Sacanella E. (2018). Nutrition and cardiovascular health. Int. J. Mol. Sci..

[23] Kocanda L., Schumacher T.L., Plotnikoff R.C., Whatnall M.C., Fenwick M., Brown L.J., Collins C.E. (2023). Effectiveness and reporting of nutrition interventions in cardiac rehabilitation programmes: A systematic review. Eur. J. Cardiovasc. Nurs..

[24] British Heart Foundation (2019). National Audit of Cardiac Rehabilitation (NACR) Report 2019-BHF.

[25] Ma Y., Olendzki B.C., Pagoto S.L., Merriam P.A., Ockene I.S. (2010). What are patients actually eating: The dietary practices of cardiovascular disease patients. Curr. Opin. Cardiol..

[26] Melia A.A., Lowe N.M., Sinclair J.K., Dillon S.A. (2016). Evaluation of nutritional knowledge, understand and practice of patients who attend a cardiac rehabilitation program in Preston. Proc. Nutr. Soc..

[27] Chaiyasoot K., Sarasak R., Pheungruang B., Dawilai S., Pramyothin P., Boonyasiri A., Batterham R.L. (2018). Evaluation of a 12-week lifestyle education intervention with or without partial meal replacement in Thai adults with obesity and metabolic syndrome: A randomised trial. Nutr. Diabetes.

[28] Moher D., Hopewell S., Schulz K.F., Montori V., Gøtzsche P.C., Devereaux P.J., Altman D.G. (2012). CONSORT 2010 explanation and elaboration: Updated guidelines for reporting parallel group randomised trials. Int. J. Surg..

[29] Sinclair J., Bottoms L., Dillon S., Allan R., Shadwell G., Butters B. (2022). Effects of montmorency tart cherry and blueberry juice on cardiometabolic and other health-related outcomes: A three-arm placebo randomized controlled trial. Int. J. Environ. Res. Public Health.

[30] Makai P., IntHout J., Deinum J., Jenniskens K., Wilt G.J.V.D. (2017). A network meta-analysis of clinical management strategies for treatment-resistant hypertension: Making optimal use of the evidence. J. Gen. Intern. Med..

[31] British Heart Foundation (2012). So You Want to Lose Weight for Good?. http://www.bhf.org.uk/publications/view-publication.aspx?ps=1000807.

[32] Lee C.L., Wang J.S. (2020). Systolic blood pressure trajectory and cardiovascular outcomes: An analysis using data in the Systolic Blood Pressure Intervention Trial. Int. J. Clin. Pract..

[33] Czernichow S., Kengne A.P., Huxley R.R., Batty G.D., De Galan B., Grobbee D., ADVANCE Collaborative Group (2011). Comparison of waist-to-hip ratio other obesity indices as predictors of cardiovascular disease risk in people with type-2 diabetes: A prospective cohort study from, ADVANCE. Eur. J. Prev. Cardiol..

[34] Guerrero-Romero F., Simental-Mendía L.E., González-Ortiz M., Martínez-Abundis E., Ramos-Zavala M.G., Hernández-González S.O., Rodríguez-Morán M. (2010). The product of triglycerides and glucose, a simple measure of insulin sensitivity. Comparison with the euglycemic-hyperinsulinemic clamp. J. Clin. Endocrinol. Metab..

[35] O’Brien E. (2003). European Society of Hypertension Working Group on Blood Pressure Monitoring: European Society of Hypertension recommendations for conventional, ambulatory and home blood pressure measurement. J. Hypertens..

[36] Pickering T.G., Hall J.E., Appel L.J., Falkner B.E., Graves J., Hill M.N., Roccella E.J. (2005). Recommendations for blood pressure measurement in humans and experimental animals: Part 1: Blood pressure measurement in humans: A statement for professionals from the Subcommittee of Professional and Public Education of the American Heart Association Council on High Blood Pressure Research. Circulation.

[37] Martínez-González M.A., García-Arellano A., Toledo E., Salas-Salvado J., Buil-Cosiales P., Corella D., PREDIMED Study Investigators (2012). A 14-item Mediterranean diet assessment tool and obesity indexes among high-risk subjects: The PREDIMED trial. PLoS ONE.

[38] Schröder H., Fitó M., Estruch R., Martínez-González M.A., Corella D., Salas-Salvadó J., Covas M.I. (2011). A short screener is valid for assessing Mediterranean diet adherence among older Spanish men and women. J. Nutr..

[39] Sofi F., Cesari F., Abbate R., Gensini G.F., Casini A. (2008). Adherence to Mediterranean diet and health status: Meta-analysis. Br. Med. J..

[40] Sinclair J., Stainton P., Dillon S., Taylor P.J., Richardson C., Bottoms L., Allan R. (2022). The efficacy of a tart cherry drink for the treatment of patellofemoral pain in recreationally active individuals: A placebo randomized control trial. Sport. Sci. Health.

[41] Lucassen D.A., Willemsen R.F., Geelen A., Brouwer-Brolsma E.M., Feskens E.J. (2021). The accuracy of portion size estimation using food images and textual descriptions of portion sizes: An evaluation study. J. Hum. Nutr. Diet..

[42] Lowe N.M., Qualter P., Sinclair J.K., Gupta S., Zaman M. (2023). School Feeding to Improve Cognitive Performance in Disadvantaged Children: A 3-Arm Parallel Controlled Trial in Northwest Pakistan. Nutrients.

[43] Wright J.T., Whelton P.K., Reboussin D.M. (2016). A randomized trial of intensive versus standard blood-pressure control. N. Engl. J. Med..

[44] Miller M., Stone N.J., Ballantyne C., Bittner V., Criqui M.H., Ginsberg H.N., Pennathur S. (2011). Triglycerides and cardiovascular disease: A scientific statement from the American Heart Association. Circulation.

[45] Talayero B.G., Sacks F.M. (2011). The role of triglycerides in atherosclerosis. Curr. Cardiol. Rep..

[46] Snetselaar L.G., de Jesus J.M., DeSilva D.M., Stoody E.E. (2021). Dietary guidelines for Americans, 2020–2025: Understanding the scientific process, guidelines, and key recommendations. Nutr. Today.

[47] Aengevaeren V.L., Mosterd A., Bakker E.A., Braber T.L., Nathoe H.M., Sharma S., Eijsvogels T.M. (2023). Exercise volume versus intensity and the progression of coronary atherosclerosis in middle-aged and older athletes: Findings from the MARC-2 study. Circulation.

[48] Pena-Hernandez C., Nugent K., Tuncel M. (2020). Twenty-four-hour ambulatory blood pressure monitoring. J. Prim. Care Community Health.

